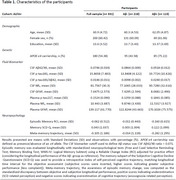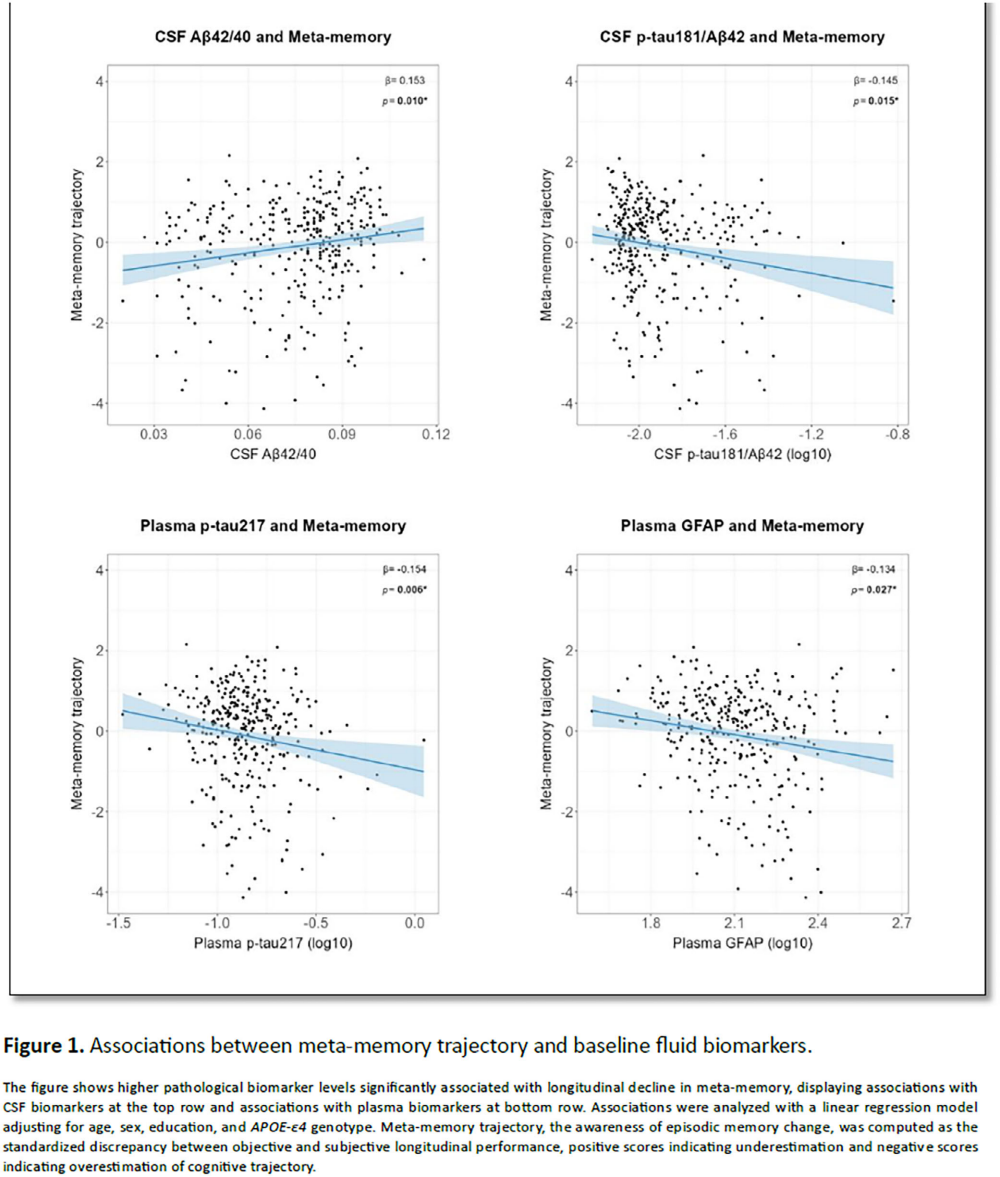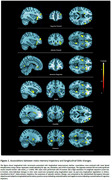# Diagnostic blind spot in clinical manifestations: anosognosia trajectory in preclinical Alzheimer's is linked to core pathophysiology, astrocytic reactivity, and structural changes in cognitive control and self‐referential brain regions

**DOI:** 10.1002/alz70857_101106

**Published:** 2025-12-24

**Authors:** David López‐Martos, Raffaele Cacciaglia, Marc Suárez‐Calvet, Armand González Escalante, Marta Milà‐Alomà, Carolina Minguillón, Clara Quijano‐Rubio, Gwendlyn Kollmorgen, Henrik Zetterberg, Kaj Blennow, Juan Domingo Gispert, Oriol Grau‐Rivera, Gonzalo Sánchez‐Benavides, ALFA study

**Affiliations:** ^1^ Barcelonaβeta Brain Research Center (BBRC), Pasqual Maragall Foundation, Barcelona, Spain; ^2^ Hospital del Mar Research Institute (IMIM), Barcelona, Spain; ^3^ Centro de Investigación Biomédica en Red de Fragilidad y Envejecimiento Saludable (CIBERFES), Madrid, Spain; ^4^ Hospital del Mar Research Institute, Barcelona, Spain; ^5^ Hospital del Mar Research Institute, Barcelona, Barcelona, Spain; ^6^ Servei de Neurologia, Hospital del Mar, Barcelona, Spain; ^7^ Universitat Pompeu Fabra, Barcelona, Spain; ^8^ Department of Radiology and Biomedical Imaging, University of California, San Francisco, San Francisco, CA, USA; ^9^ Department of Veterans Affairs Medical Center, Northern California Institute for Research and Education (NCIRE), San Francisco, CA, USA; ^10^ Barcelonaβeta Brain Research Center, Barcelona, Spain, Barcelona, Spain; ^11^ Roche Diagnostics International Ltd., Rotkreuz, Switzerland; ^12^ Roche Diagnostics GmbH, Penzberg, Germany; ^13^ Hong Kong Center for Neurodegenerative Diseases, Hong Kong, Science Park, China; ^14^ Wisconsin Alzheimer's Disease Research Center, University of Wisconsin‐Madison, School of Medicine and Public Health, Madison, WI, USA; ^15^ Department of Neurodegenerative Disease, UCL Institute of Neurology, Queen Square, London, United Kingdom; ^16^ UK Dementia Research Institute, University College London, London, United Kingdom; ^17^ Clinical Neurochemistry Laboratory, Sahlgrenska University Hospital, Gothenburg, Sweden; ^18^ Department of Psychiatry and Neurochemistry, Institute of Neuroscience and Physiology, The Sahlgrenska Academy at the University of Gothenburg, Mölndal, Västra Götalands län, Sweden; ^19^ Clinical Neurochemistry Laboratory, Sahlgrenska University Hospital, Mölndal, Västra Götalands län, Sweden; ^20^ Department of Psychiatry and Neurochemistry, University of Gothenburg, Gothenburg, Sweden; ^21^ AstraZeneca, Barcelona, Spain; ^22^ Centro de Investigación Biomédica en Red de Fragilidad y Envejecimiento Saludable (CIBERFES), 28089, Madrid, Spain

## Abstract

**Background:**

Anosognosia, lack of awareness of clinical impairment, is a common manifestation in Alzheimer's disease (AD). This phenomenon is linked to AD neuropathology and dementia progression, but this is not acknowledged in Mild Cognitive Impairment (MCI) diagnostic criteria. Instead, only Subjective Cognitive Decline (SCD) is considered, even though self‐reported SCD is not specific to AD and may be already absent in MCI patients. Anosognosia might be overlooked in clinical practice, and its neuropathological underpinnings remain unknown. This study examined the longitudinal relationship between multimodal AD biomarkers and meta‐cognitive changes in cognitively unimpaired (CU) individuals at risk for AD dementia.

**Methods:**

This research included 331 CU participants with early AD biomarker profiles [Table 1; ALFA+ cohort]. Study measures involved baseline Cerebrospinal Fluid (CSF) and plasma biomarkers, as well as three‐year longitudinal Magnetic Resonance Imaging (MRI) and neuropsychological evaluation. Episodic memory was evaluated with a composite of standardized tests, computing a longitudinal Reliable Change Index (RCI). Self‐perceived cognitive decline was measured at follow‐up with the SCD‐Questionnaire, providing a retrospective subjective index matching longitudinal time interval. Awareness of episodic memory change, meta‐memory trajectory, was computed as the standardized discrepancy between objective and subjective longitudinal performance. The relationship of baseline CSF (Aβ42/40, *p*‐tau181, *p*‐tau181/Aβ42, NfL, GFAP) and plasma (*p*‐tau217, NfL, GFAP) biomarkers with meta‐memory trajectory was evaluated using linear regression. Grey Matter volume (GMv) changes were evaluated using Longitudinal Voxel Based Morphometry.

**Results:**

Meta‐memory decline was associated with lower CSF Aβ42/40 (β=0.153, *p* = 0.010) and higher CSF *p*‐tau181/Aβ42 (β=‐0.145, *p* = 0.015), but not with CSF *p*‐tau181, CSF NfL, or CSF GFAP. It was also associated with higher plasma *p*‐tau217 (β=‐0.154, *p* = 0.006) and plasma GFAP (β=‐0.134, *p* = 0.027), but not with plasma NfL (Figure 1). Meta‐memory decline was associated with increased GMv in the frontal cortex, anterior cingulate cortex, insula, and striatum (Figure 2).

**Conclusions:**

These results demonstrated that anosognosia's trajectory is linked to AD pathophysiology and structural brain changes in pre‐MCI stages, underscoring the need for early detection methods beyond self‐reported symptoms. Identifying high‐risk individuals who may delay or not seek medical attention is critical for implementing objective diagnostics and prevention, still current criteria fail to explicitly address this challenge.